# Upper secondary school students’ compliance with two Internet-based self-help programmes: a randomised controlled trial

**DOI:** 10.1007/s00787-017-1035-6

**Published:** 2017-08-03

**Authors:** Carl Antonson, Frida Thorsén, Jan Sundquist, Kristina Sundquist

**Affiliations:** 1 Department of Clinical Sciences Malmö, Center for Primary Health Care Research, Lund University, Malmö, Sweden; 2 Clinical Research Centre (CRC), Building 28, Floor 11, Skåne University Hospital, Jan Waldenströms gata 35, 205 02 Malmö, Sweden

**Keywords:** Adolescence, Compliance, Internet, Mindfulness, Psychiatric symptoms, Psychological stress

## Abstract

Psychiatric symptoms and stress are on the increase among Swedish adolescents. We aimed to study the potential effect and feasibility of two Internet-based self-help programmes, one mindfulness based (iMBI) and the other music based in a randomised controlled trial that targeted adolescents. A total of 283 upper secondary school students in two Swedish schools were randomised to either a waiting list or one of the two programmes, on their own incentive, on schooltime. General psychiatric health (Symptoms Checklist 90), sleep quality (Pittsburgh Sleep Quality Index), and perceived stress (Perceived Stress Scale) were assessed before and after the interventions. In total, 202 participants answered the questionnaires. Less than 20 logged into each intervention and only 1 performed a full intervention (iMBI). No significant differences in any of the scales were found between those who logged in and those who did not. The potential effect of Internet-based self-help programmes was not possible to examine due to low compliance rates. Adolescents seem to have a very low compliance with Internet-based self-help programmes if left to their own incentive. There were no associations between the psychiatric and stress-related symptoms at baseline and compliance in any of the intervention groups, and no evidence for differences in compliance in relation to the type of programme. Additional studies are needed to examine how compliance rates can be increased in Internet-based self-help mindfulness programmes in adolescents, as the potentially positive effects of mindfulness are partly related to compliance rates.

**Electronic supplementary material:**

The online version of this article (doi:10.1007/s00787-017-1035-6) contains supplementary material, which is available to authorized users.

## Background

Psychiatric symptoms and perceived stress have increased among Swedish adolescents in recent decades [1–3]. Furthermore, there is evidence of significant associations between stress and psychopathology in children and adolescents from both cross-sectional [4, 5] and longitudinal studies [6–9]. Psychiatric symptoms and stress in adolescents can result in negative effects on health, well-being, and academic achievement [10], and seem to be independent of both the academic proficiency and urbanicity of the school [11]. In addition, there is a well-established association between reduced sleep quality and psychiatric symptoms in adolescents. Low sleep quality in adolescents is associated with behavioural problems and impairments in cognitive functioning and mood [12–14], which partly could explain the suggested increase in psychiatric symptoms and stress in Swedish adolescents over time [1, 15].

Internet-based psychotherapy (iPT) seems to be a promising treatment alternative in targeting psychiatric health problems; iPTs are relatively easy to access and could provide therapy to many people at a low cost [16]. This form of therapy may be particularly suitable for adolescents, since young people in general are familiar with using the Internet and it could also help many adolescents who refrain from seeking help in regular health care [17]. The effects of, as well as the compliance to, iPTs in adolescents remain, however, largely unstudied as very few studies have been based on younger participants [18].

Mindfulness-based interventions (MBIs) have been adapted to the Internet as a vehicle of delivery and could therefore be suitable for adolescents. MBIs are well-renowned and well-validated psychotherapies [19–28], and a meta-analysis in non-clinical settings concluded that mindfulness-based stress reduction (MBSR) can reduce stress levels in healthy people [29]. A recent randomised controlled trial (RCT) in Sweden was conducted on adults with anxiety, depression, and stress and adjustment disorders treated in primary health care. The RCT compared group-based mindfulness therapy with individual-based cognitive behavioural therapy (CBT) and found that both therapies resulted in significant decreases in psychiatric symptoms [30]. Mindfulness-based therapies can also be combined with CBT, i.e. mindfulness-based cognitive therapy (MBCT) [31].

Two recent review articles concerning mindfulness on adolescents concluded that it holds promise for students, in terms of improving cognitive performance and resilience to stress [32, 33]. Another study concluded that there are few possibilities to deliver MBIs in schools as few teachers, or other school personnel, are able to provide MBIs to the students [34]. The use of Internet-based MBIs (iMBI) may therefore be promising in terms of accessibility [16]. Several studies on adults have shown promise in using the Internet as a vehicle of psychotherapy delivery [35–40]. A narrative review concluded that iMBIs are feasible in terms of effectiveness, acceptability, and economy, and that further research examining different modes of iMBIs is warranted [41]. To our knowledge, no iMBI study on the potential effects as well as compliance has been carried out on adolescents. However, an Australian qualitative study has proposed iMBI as something desired by many adolescents [42].

Some previous studies have examined the potential effects of iMBI as well as the compliance in adults. The included clinical diagnoses and outcomes were relapse of major depression [39] and depression, anxiety and stress and adjustment disorders [35, 36, 38, 40, 43]. In general, these studies have found positive effects of iMBI, although the compliance has varied.

The first aim of this study was to investigate whether there is an effect of iMBI, delivered as an Internet-based self-help programme, on psychiatric and stress-related symptoms in adolescents. The second aim was to investigate the feasibility, in terms of compliance, of the intervention. The third aim was to investigate whether there is any association between psychiatric and stress-related symptoms at baseline and compliance to the intervention in the adolescents.

## Methods

### Study population

In the process of choosing the population, we opted for two different schools in terms of academic performance and urbanicity, to obtain an adequate sample that could be considered relatively representative of Swedish upper secondary school students. This selection is more thoroughly described in the baseline cross-sectional study [11], which represents a larger randomised, controlled, single-blinded study (CHAMPS) aimed to investigate whether iMBI could be used to prevent psychiatric and stress-related symptoms.

All Swedish-speaking students enrolled at the schools (adolescents aged 15–19 years) received information, initially through personal letters sent to their home addresses, and thereafter lectures in the schools (10-min oral information from the researchers in groups of 10–100 students with the possibility to ask questions).

We had three inclusion criteria: to be able to read and understand Swedish, to have an e-mail address, and to be willing to participate in an 8-week Internet-based self-help programme. A total of 1404 students were attending the two schools during the study period. The Swedish-speaking inclusion criterion excluded one subject, leaving 1403 possible participants. The second inclusion criterion did not lead to exclusion of any potential participants and the third criterion, willingness to participate in a stress reduction intervention, left 283 individuals all of whom gave written informed consent. The 283 participants were numbered consecutively after the arrival of their written consent form and were divided into seven groups, based on the three grades (first to third year of upper secondary school) and sex, to acquire all grades and both sexes in all intervention groups. This led to the construction of the following seven groups: first-grade male, first-grade female, second-grade male, second-grade female, third-grade male, third-grade female, and others. Others were classified as participants where grade and/or sex was missing. After being categorised in this way, the individuals in each of the seven groups were computer randomised into three intervention groups (A–C) by a statistician, i.e. each individual was assigned the letter A, B or C. The three treatment groups were iMBI, Internet-based music therapy (iMT), and waiting list. The initial seven groups were only created for randomisation purposes.

Of the 283 participants who gave written consent, 202 students—142 female, 50 male, and 10 where the information on sex and/or school was missing—also answered the Web-based questionnaires. The more rural school provided 45 students and the remaining 147 participants came from the more urban school. The mean age was 16.9 years and the median age 17 years (range 15–19 years).

Both intervention groups represented Internet-based self-help programmes and the coordinator at the institution handled all contact with the study subjects. This was to preserve the single-blindness of the intervention to the researchers. The participants were allowed by the schools to do a 10-min daily intervention during lesson time and were asked to do at least one intervention on each school day. There were no reminders given to the students, i.e. they had to participate in their appointed intervention on their own incentive. The 8-week interventions commenced on March 15, 2012. The participants could, via e-mail, contact two of the co-authors of this study (CA or FT), who are also physicians, if they felt that their psychiatric health deteriorated during the interventions.

Power was calculated for the main outcome of the study, improvement in global severity index (GSI) (see below) with the conventional values for significance level of 0.05 (*α*), and for power of 0.80 (*β*). With three groups to be compared, we used a one-way ANOVA pairwise two-sided equality calculation. We estimated a priori that with a population sample of 73 in each group, the GSI in the iMBI group would differ significantly from the waiting list group. This was based on a face-to-face mindfulness study in adults where GSI, our primary outcome, was measured [44]. In that study, the authors found an improvement in GSI from 0.91 (SD 0.71) to 0.53 (SD 0.51) and a median change of –43.2% after an 8-week MBSR course. According to the power calculation, we needed at least 219 participants, i.e. 73 in each group. To compensate for a presumed high pre-baseline dropout rate, we estimated the dropout rate before the first questionnaire to be 70%, based on two Swedish studies using GSI as an outcome in adolescents [45, 46]. We also made an estimation of the post-baseline dropout rate, of 44%, based on the responders that actually complied with an intervention in an adult iMBI study [43]. These assumptions led to an estimated total sample size of 1140 in the present study. We judged that the population of 1403 in the two chosen schools was adequate to address the study aims.

A recent systematic review of 36 studies [47] listed the different definitions/measures of dropout and engagement. As it does not seem as if a consensus has been reached on what terminology is most appropriate, we discriminated those who logged in with those who did not as follows: ‘logged in’ and ‘not logged in’. We believe that this represents the most accurate description of those students who enrolled in our study [47, 48].

### Interventions

The two active interventions were Internet-based self-help programmes that were completely computerised without any human interference. They were based on modules of approximately 10 min and contained both video and audio material, but the focus was on the latter. Both interventions logged compliance in terms of log in identification, time at log in, and fulfilment of the session. They were accessible by any device that connects to the Internet, such as computers, smartphones, and tablets. All groups, including the waiting list group, got access to both interventions after all post-intervention questionnaires were completed. No further follow-up was performed.

### Mindfulness-based intervention (iMBI)

The iMBI (In Swedish: *Mindfulness Grundkurs 2.0* from Mindfulnesscenter AB, Sweden) was designed by Dr. Ola Schenström, a family physician and renowned national expert in Sweden on clinical mindfulness meditation. The programme is an 8-week course consisting of sessions of 10 min of mindfulness meditation twice daily, 6 days a week. The modules consist of standard mindfulness meditation techniques, such as body scan and mindfulness of breath, and other perceptions [49], and could be defined as an intervention based on mindfulness training [31]. The intervention incorporates elements from both MBSR [50] and more cognitively oriented parts from MBCT [51]. An iMBI, in a study on anxiety in adults, has used the same platform and guided meditations, but with cognitive material focused on anxiety [40]. For our study, a complete intervention was defined as at least 40 sessions, which is lower than the original programme but was deemed appropriate by the constructor of the programme.

### Music therapy intervention (iMT)

We chose iMT as the active control to iMBI, as one meta-analysis on music therapy showed a good effect in stress reduction from listening to music both in itself and in combination with music-assisted relaxation techniques, and concluded that the best effect is on adolescents [52]. Listening to music is also something that adolescents are generally interested in and tend to do, of their own free will, when feeling stressed or emotionally challenged [53]. Furthermore, a small study on adolescents targeting depression with music therapy, which included active participation with instruments and interpretation in terms of painting, showed promising results [53]. In addition, the partial similarity between the two Internet-based self-help programmes makes these two groups comparable. For the present study, a complete intervention was defined as at least 40 sessions.

The iMT, *Musikintervention*, was designed with the aid of Professor Björn Ejdemo, MD, and visiting professor of music at the Australian National University, who made a preliminary selection of pieces of music. Per Vegfors, MD, specialist in child and adolescent psychiatry, assessed the appropriateness of the music from an adolescent psychiatric perspective. A total of ten non-vocal classical music pieces accessible on YouTube were chosen that met the criteria of (1) accessibility, i.e. being relatively easy to listen to for an untrained ear, (2) being of approximately 10 min in duration, and (3) recognisable as being calming or soothing. The programme Musikintervention (Paxx Media AB, Sweden) used streamed music videos from YouTube. See appendix for list of music and interpretations.

### Questionnaires

We used three well-defined, well-validated, and reliable psychometric tests, before and after the intervention, that had previously been used on adolescents: the Symptoms Checklist 90 (SCL-90) [54], the Perceived Stress Scale (PSS-14) [55], and the Pittsburgh Sleep Quality Index (PSQI) [56]. The combination of scales was chosen to give an insight into the students’ perceived stress (PSS-14) and likely outcomes of that stress, expressed as low-quality sleep (PSQI) and increased general psychiatric symptoms (SCL-90), as previous studies have shown bi-directionally stress-associated impairments in psychiatric symptoms and sleep [11]. The time required to fill in the questionnaires was adjusted to avoid questionnaire fatigue and keep good test–retest reliability.

### Symptoms Checklist 90 (SCL-90)

To measure general psychiatric symptoms, we used the 90-item SCL-90, which uses a five-point Likert scale to assess overall psychiatric symptoms, including somatisation. The main outcome of the scale is the GSI, which is calculated as the total sum of the weights for each individual item divided by the total number of questions answered (with a minimum answer rate of 80%). The SCL-90 is commonly used in psychiatric evaluations and has a subscale measuring somatisation [54]. The SCL-90 has satisfactory internal consistency reliability [57, 58] and test–retest reliability over both a week [54] and 10 weeks [58]. The latter study also showed a good test–retest reliability coefficient for the GSI. The validity of the scale has been confirmed with regard to internal structure, factorial invariance, and convergent–discriminant validity [54, 57, 59, 60].

### Perceived Stress Scale (PSS-14)

For measurement of perceived stress, we used the widely accepted 14-item PSS, constructed as a five-point Likert scale [55, 61]. The PSS only focuses on how the individual experiences the stressors and not on their magnitude. The PSS-14 scale has been shown to have good internal consistency and reliability (Cronbach’s alpha 0.75–0.89), and to have good test–retest reliability over 2 days to 4 weeks. It has been empirically validated in college students [62]. Measurements of perceived stress have higher ecological validity than physiological response parameters, self-report of psychiatric symptoms, and behavioural changes and stressors, such as major life changes [63].

### Pittsburgh Sleep Quality Index (PSQI)

We used the PSQI [56] to measure sleep quality. This index is an algorithm that calculates sleep quality based on nine parameters (one of which is divided into eight sub-items) and results in a numerical value with a cutoff level for low-quality sleep of more than five points. The PSQI has been shown to have good test–retest reliability for both the global score and the subscores in both short-term (2 days) and long-term (1–2 months) follow-ups, with an overall Cronbach’s alpha of 0.87. The PSQI has also shown good correlations with sleep logs and lower, but significant, correlations with polysomnography, confirming its validity [64].

The tests were sent via e-mail to the address given by each student when signing the informed consent document. The students were able to answer the tests when it suited them during a window of ten consecutive days. All questionnaires were handled by the Internet-based survey programme Inquisite Survey System (Inquisite Inc., Copenhagen, Denmark). Data were stored unidentified to guarantee privacy and blinding.

### Statistical analysis

To examine aim 1, i.e. to investigate whether there was a potential effect of the Internet-based self-help programmes on psychiatric and stress-related symptoms assessed before and after the intervention, we aimed to compare the scores of the different scales before and after the intervention among the active participants. We also used Student’s *t* test to investigate whether the three groups differed at baseline. These comparisons were performed to check whether the randomisation procedure was successful. To examine aim 2, i.e. to investigate the feasibility in terms of compliance to the interventions with the two Internet-based self-help programmes, we used a CONSORT flowchart to display the dropout rates at the different steps of the study [65]. The comparison in dropout rate between the two intervention groups (iMBI and iMT) was done using Fisher’s two-tailed exact test calculated with the number of participants that were randomised to either intervention and the participants that finished at least one session of either intervention. The Fisher’s two-tailed exact test was used as we intended to analyse whether the relatively small samples deviated from the null hypothesis that there was a difference between the dropout rates between iMBI and iMT. To examine aim 3, i.e. to investigate whether there is any association between compliance to the intervention and psychiatric and stress-related symptoms at baseline, we compared the participants that logged in and completed at least one session and those who did not log into the self-help programme. We compared whether there was a difference in psychiatric and stress-related symptoms between those two groups, separately in each intervention group (iMBI and iMT). We used a scatter plot to check whether the distribution was skewed and used the non-parametric Wilcoxon rank-sum test if the distribution was skewed and the parametric Student’s *t* test when the data were normally distributed.

Statistical analysis was done using Stata IC 12 (Stata Corp, Texas, USA).

## Results

**Fig. 1:**
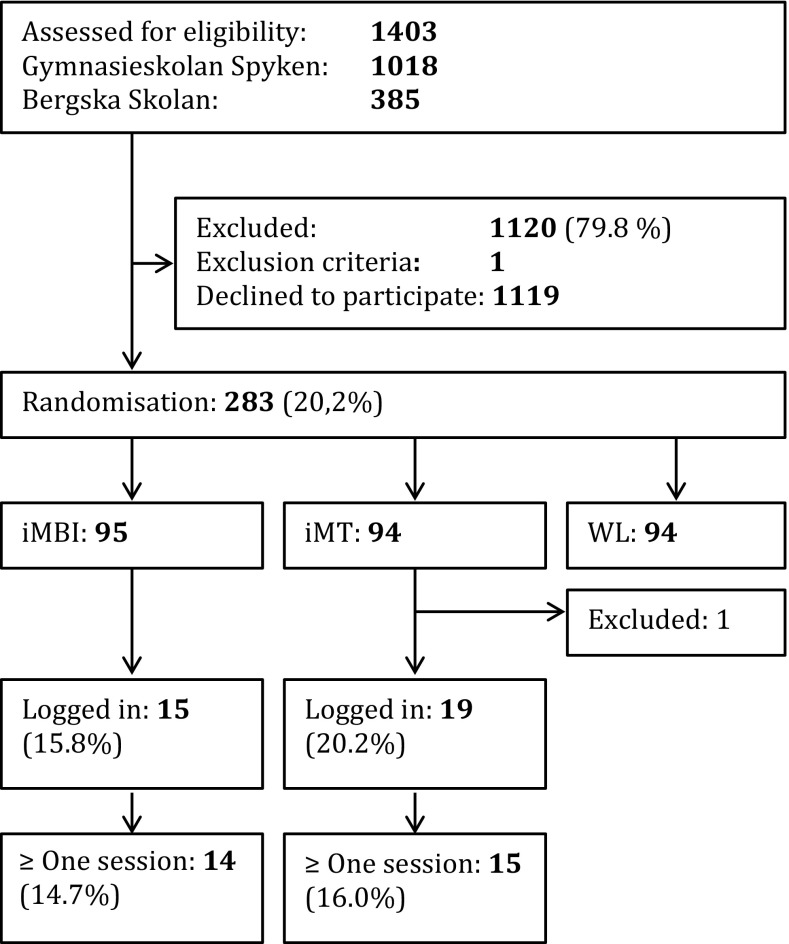
CONSORT flowchart of the study in terms of compliance to the interventions. The participant who logged into both interventions was excluded after the randomisation. The percentages in the intervention groups are the proportions of the randomised participants assigned to each intervention. Interventions: *iMBI* Internet-based mindfulness-based intervention, *iMT* Internet-based music therapy, and *WL* waiting list

283 students were randomised into the three groups of the study: iMBI (*n* = 95), iMT (*n* = 94), or waiting list (*n* = 94); 1119 eligible candidates declined participation and 1 was excluded due to the language exclusion criterion. The randomisation worked well, as no significant differences were found between the three groups before the intervention in any of the scales. Of the 283 participants, 1 logged into both interventions and was excluded from the analysis. Of the 282 remaining students, 15 (15.8%) logged into the mindfulness intervention and 19 (20.4%) to the music intervention. Of the 19 persons who logged into the iMT, 4 did not complete any session, which was defined as playing at least 50% of the pieces of music. All but one person logging into the iMBI fulfilled at least one session. The flowchart is shown in Fig. 1. The distribution curve of sessions is shown in the supplementary Figure.

Table 1 shows the comparisons between those who logged in and completed at least one session and those who did not log into the self-help programmes. No significant difference in any of the scales was found between those who logged in and completed at least one session and those who did not log into the self-help programmes.

**Table 1:** Differences between those who logged in to at least one session and those who did not log into the self-help programmes (i.e. *iMBI* Internet-based mindfulness-based intervention and *iMT* Internet-based musical therapy) in general psychiatric symptoms (GSI from Symptoms Checklist-90), perceived stress (*PSS* Perceived Stress Scale-14), and sleep quality (*PSQI* Pittsburgh Sleep Quality Index)

Scale	IMBI			IMT		
	Logged in	Not logged in	*p*	Logged in	Not logged in	*p*
GSI *n*	13	63		15	56	
GSI mean (SD)	0.96 (0.46)	0.84 (0.57)		0.78 (0.53)	0.83 (0.47)	
GSI CI	0.68–1.24	0.70–0.98	0.30^a^	0.48–1.07	0.71–0.96	0.59^a^
PSS *n*	15	56		14	56	
PSS mean (SD)	29.4 (7.43)	30 (8.52)	0.41^b^	30.7 (9.49)	28.7 (6.96)	0.82^b^
PSS CI	25.3–33.5	27.7–32.3		25.2–36.2	26.8–30.5	
PSQI *n*	14	57		13	55	
PSQI mean (SD)	5.93 (0.89)	6.93 (0.42)		6.69 (3.20)	6.09 (2.53)	
PSQI CI	4.0–7.86	6.09–7.77	0.40^a^	4.76–8.62	5.41–6.77	0.65^a^

^a^ Skewed distribution—Wilcoxon rank sum

^b^ Normal distribution—Student’s *t* test

Fisher’s exact test (two-tailed) gave a *p* value of 0.842 on comparing the number of participants who did at least one session of either intervention (iMBI or iMT), excluding the person who logged into both interventions.

The data below are only descriptive, since the compliance rate to the interventions was low. The compliance to the study protocol was 72% at baseline.

The only participant who did a complete intervention, defined as at least 40 sessions, performed 42 iMBI sessions and decreased the GSI by 31% (from 0.80 to 0.55). The same person decreased in PSQI from 8 to 7 and in PSS from 29 to 23. As regards the participants completing at least ten sessions (i.e. 25%), there were four in the iMBI group, three of whom answered the post-intervention questionnaires. In the iMT group, only one participant completed at least 25% of the sessions. The iMBI group decreased their mean GSI from 0.85 to 0.63 (SD 0.13 and 0.19, respectively). Their PSS decreased from 29.0 to 26.3 (SD 6.0 and 5.8, respectively). PSQI decreased from 9 to 8 (SD 4.6 and 5.5, respectively). The only iMT participant fared worse, with GSI increasing from 0.68 to 0.90, PSQI increasing from 6 to 7, but with PSS decreasing from 26 to 24.

None of the participants used the possibility to contact a physician via e-mail.

## Discussion

Our main finding in this RCT is that the compliance rates of the two Internet-based self-help programmes were very low in adolescents (aim 2). This in itself is of interest, although the limited compliance did not allow us to investigate our first aim due to insufficient statistical power. However, the study provides useful information for the planning of future studies. We found no association between psychiatric and stress-related symptoms and compliance in any of the intervention groups (aim 3).

Our second aim, to determine feasibility in terms of compliance to the intervention, shows that the feasibility in conducting Internet-based self-help programmes in mindfulness may be limited in adolescents and that more research is needed on why the compliance is low in non-clinical samples. A limited compliance to an intervention may occur in several steps and present as failure to engage in the intervention or drop out from the protocol. In the present study, the low compliance after the randomisation/baseline assessment mainly occurred in two steps. First, a limited number of the adolescents actually logged in: only 15 of the 95 and 19 of the 94 adolescents logged into the iMBI and iMT, respectively. Second, the adolescents who actually logged in only completed a limited number of sessions and only one adolescent completed all 40 sessions.

The positive effects of mindfulness training are partly related to compliance rates. A recent meta-analysis on face-to-face MBIs in adolescents showed a positive correlation between minutes of mindfulness training and effect sizes [32]. Kuyken et al. reported that at least 50% attendance at the eight sessions of MBCT is considered necessary to receive an adequate treatment dose [66]. Thus, more knowledge is needed on the potential causes behind low compliance as well as methods to increase compliance with iMBI.

### Potential causes behind and ways to overcome low compliance rates

According to Christensen et al. [67], there are three general approaches to investigate compliance in Internet-based interventions for anxiety and depression; these approaches can also be used in other types of patients and interventions. The first involves the examination of correlations to personality, demographic, and service delivery factors. The second includes the use of post-test qualitative investigations to obtain retrospective analyses of peoples’ perceptions of trial participation and barriers to the use of the Internet intervention, and the third involves experimental manipulation of variables thought to change compliance. The first approach has shown that lower compliance is associated with higher levels of emotional distress [67], certain types of conditions (higher compliance in chronic headache patients than in weight control patients), and male sex combined with lower GSI in cardiac patients [68]. Kabat-Zinn and Chapman-Waldrop found, however, no association between compliance and GSI in referred somatic and psychiatric patients [68]. Compliance rates may decrease in Internet-based [18, 69], longer, and preventive (rather than treatment) interventions in healthy population-based samples [70]. We found no evidence of an association between compliance rates and psychiatric and stress-related symptoms in our study population of non-clinical Swedish adolescents, which is in accordance with the study by Kabat-Zinn and Chapman-Waldrop who concluded that “there was no indication that severity of symptoms or illness affected outcome in terms of programme completion”. It is possible that feelings of shame or embarrassment could have been behind our students’ reluctance to log into the interventions during class time, although it also was possible for the students to log in outside class time. Another possibility is that some students may have found the intervention to be anxiety inducing, as previous studies have shown that emotional distress [67] decreases compliance.

The second and third approaches for compliance investigation according to Christensen et al. were not included as part of our study. Anecdotally, some students found the intervention ‘boring’ and others said that they did not complete the intervention due to lack of time. A teacher, school nurse, and school counsellor were of the opinion that stress, due to school issues, made the students less compliant to such interventions. Both students and personnel mentioned that stress interventions should be part of the curriculum to increase compliance and decrease stress. In an RCT conducted in Belgian schools, where mindfulness classes were part of the curriculum, the compliance rate was 85% [71].

Previous studies have examined how compliance to iPTs can be increased. For example, an Australian study found that compliance to iCBT in adults could be increased with e-mail reminders [69]. Some studies have found that guidance by trained psychotherapists augments the participation rate [72], while other studies, including university students, have found no such effect [73]. Material incentives have been shown to enhance compliance in children and adolescents as well as adults [74–77].

These potential causes and potential ways to increase compliance (see above) may be more or less salient during the different phases of the intervention, i.e. there may be different types of resistance towards entering and staying in the programme or, in this case, to log in or not log into the Internet-based self-help programme. For example, embarrassment and shame, gender, or a wish to belong to a specific intervention may deter students from logging in, whereas at later stages in the study other factors may lead to dropouts, such as psychological distress/anxiety, programme content, and lack of incentives. Some factors may lead to low compliance during the entire intervention, such as lack of time and stress, which may call for the inclusion of stress interventions into the curriculum.

### Limitations and strengths

There are several limitations with the present study. We had no strategy to address any low compliance in the present study apart from our attempts to increase the sample size. However, an important aim was to study the potential feasibility of the two Internet-based self-help programmes in an RCT that targeted students in their adolescence, which represents a novel contribution. The low compliance rate did not allow for sufficient power to investigate our primary aim regarding the potential effect of the two interventions. In addition, we have only anecdotal information on why the adolescents dropped out. The use of questionnaires could have led to self-report bias, but questionnaires have been found to provide reliable data in Nordic adolescents [78]. Another limitation of this intervention is that it might be dubious to introduce stress management methods, such as mindfulness, with a sole focus on the individual while neglecting the overall organisation and structures in the school setting, which in itself may entail stress-inducing characteristics. Instead, a relational approach, which sees mindfulness as a socially contingent resource for communities, has been suggested as an alternative to the more common focus on the individual [79, 80]. Finally, although not being a direct limitation of the study, one could argue that all types of school interventions might compete with other important activities, such as schoolwork.

However, there are also several strengths that could be useful in future studies. For example, we included an active control group, listening to music, which is something that adolescents tend to do when feeling stressed or emotionally challenged [53]. A focus on listening is sometimes included in MBI, as in MBCT [51], and the partial similarity between the two interventions [81] makes these two groups comparable in terms of focus, although the focus in mindfulness is purposeful and without judgment. Another strength is that we evaluated the compliance in several steps [67]. The study design included reliable, well-validated questionnaires, and no adverse reactions to any of the interventions were reported. Finally, this is the first RCT that aimed to compare two different Internet-based self-help programmes in both male and female adolescents.

## Conclusions

In conclusion, the compliance rates of the two Internet-based self-help programmes were very low in adolescents, which did not allow us to investigate our primary aim. The study provides, however, useful information for the planning of future studies in adolescents. Future studies need to examine potential causes behind why compliance rates may be low in Internet-based self-help programmes and how compliance rates can be increased in adolescents, as the potentially positive effects of mindfulness programmes on psychiatric symptoms and stress most likely are related to compliance rates.

## Electronic supplementary material

**Below is the link to the electronic supplementary material.**

**Supplementary material 1 (DOCX 98** **kb)**

**Supplementary material 2 (DOCX 81** **kb)**
