# Supplementary material for: Upper secondary school students’ compliance with two Internet-based self-help programmes: a randomised controlled trial
Source: Eur Child Adolesc Psychiatry. 2017 Aug 3;27(2):191–200. doi: 10.1007/s00787-017-1035-6 (PMC5842245; doi:10.1007/s00787-017-1035-6)
Supplement: Supplementary file 1 — Supplementary material 1 (DOCX 98 kb) [file 787_2017_1035_MOESM1_ESM.docx]

| **Composer** | **Piece** | **Opus** | **Soloist** | **Conductor** | **Orchestra** | **Time** | **Youtube** |
| --- | --- | --- | --- | --- | --- | --- | --- |
| Pyotr Ilyich Tchaikovsky | Piano Concerto No. 1 | 23 | Sviatoslav Richter | Yevgeny Mravinsky | Leningrad Philharmonic Orchestra | 09:33 | http://www.youtube.com/watch?v=3oMm34x1pBg&feature=related |
| Joaquín Rodrigo | Concierto de Aranjuez, Adagio | n/a | Narciso García Yepes | Raphael Frühbeck | Frankfurt Radio Symphony Orchestra | 10:52 | http://www.youtube.com/watch?v=CY29JlyAH7c |
| Antonín Dvořák | Symphony Nr 9, movement 1 | 95 | n/a | Georg Solti | Chicago Symphony Orchestra | 10:37 | http://www.youtube.com/watch?v=m_FrPV4uNic |
| Bedřich Smetana | Má Vlast - Vltava | n/a | n/a | Ferenc Fricsay | n/a | 10:21 | <http://www.youtube.com/watch?v=-fsYPz4aWnc> |
| Ludwig van Beethoven | Violin Concerto, D-major, movement 1 | 61 | Anne-Sophie Mutter | Seiji Ozawa | Boston Symphony Orchestra | 10:01 | http://www.youtube.com/watch?v=D3zsetUus98&feature=related |
| Tomaso Giovanni Albinoni | Chaconne, G-minor | n/a | David Oistrakh | n/a | n/a | 10:15 | http://www.youtube.com/watch?v=i4B1ifcWa9o&feature=related |
| Sergey Rachmaninoff | Piano Concerto No. 2 in C-minor, Moderato | 18 | Sergey Rachmaninoff | Leopold Stokowski | Philadelphia Orchestra | 09:35 | http://www.youtube.com/watch?v=x8l37utZxMQ&feature=related |
| Maurice Ravel | Piano Concerto No 1, movement 1 | n/a | Arturo Benedetti Michelangeli | Sergiu Celibidache | London Symphony Orchestra | 09:50 | http://www.youtube.com/watch?v=f0T4rCiSZjQ&feature=related |
| Edward Elgar | Cello Concerto, movement 1 | 85 | Jacqueline du Pré | n/a | n/a | 12:58 | http://www.youtube.com/watch?v=_J-Iwtzzge8&feature=related |
| Franz Schubert | Piano Trio No 1, movement 2 | 99 | Yehudi Menuhin, Hephziba Menuhin, Maurice Gendron | n/a | n/a | 09:23 | http://www.youtube.com/watch?v=GBuHBQaYZoU&feature=related |
| Arcangelo Corelli | Concerto Grosso No. 1 in D-major | 6 | n/a | Nicholas McGegan | n/a | 11:00 | http://www.youtube.com/watch?v=oxtO9CzF7-o |
