# Supplementary material for: Upper secondary school students’ compliance with two Internet-based self-help programmes: a randomised controlled trial
Source: Eur Child Adolesc Psychiatry. 2017 Aug 3;27(2):191–200. doi: 10.1007/s00787-017-1035-6 (PMC5842245; doi:10.1007/s00787-017-1035-6)
Supplement: Supplementary file 2 — Supplementary material 2 (DOCX 81 kb) [file 787_2017_1035_MOESM2_ESM.docx]

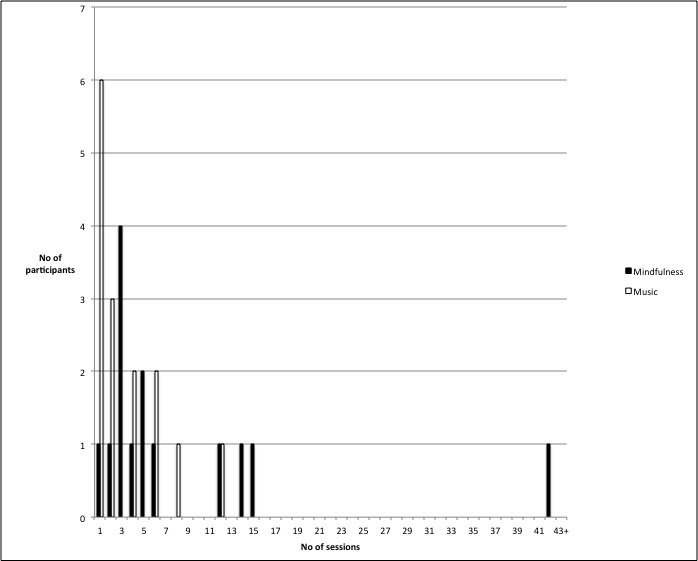


*SUPPLEMENTAL FIGURE 1. Distribution of sessions completed in the respective intervention group. Forty sessions was the complete intervention consisting of one session daily, five times a week for eight weeks. Number of participants who never did a full intervention session of 10 minutes: Mindfulness (internet-based Mindfulness Based Intervention) = 80, Music (internet-based Musical Therapy) = 78 (out of 95 and 93, respectively). The person who logged in on both interventions is excluded in the graph.*
